# Human Condensin Function Is Essential for Centromeric Chromatin Assembly and Proper Sister Kinetochore Orientation

**DOI:** 10.1371/journal.pone.0006831

**Published:** 2009-08-28

**Authors:** Alexander Samoshkin, Alexei Arnaoutov, Lars E. T. Jansen, Ilia Ouspenski, Louis Dye, Tatiana Karpova, James McNally, Mary Dasso, Don W. Cleveland, Alexander Strunnikov

**Affiliations:** 1 Unit on Chromosome Structure and Function, National Institute of Child Health and Human Development (NICHD), Laboratory of Gene Regulation and Development (LGRD), Bethesda, Maryland, United States of America; 2 Section on Cell Cycle Regulation, National Institute of Child Health and Human Development (NICHD), Laboratory of Gene Regulation and Development (LGRD), Bethesda, Maryland, United States of America; 3 Ludwig Institute For Cancer Research, Department of Cellular and Molecular Medicine, University of California San Diego, La Jolla, California, United States of America; 4 National Institute of Child Health and Human Development (NICHD), Laboratory of Functional and Molecular Imaging, Bethesda, Maryland, United States of America; 5 Fluorescence Imaging Facility, National Cancer Institute (NCI), Bethesda, Maryland, United States of America; National Cancer Institute, United States of America

## Abstract

Condensins I and II in vertebrates are essential ATP-dependent complexes necessary for chromosome condensation in mitosis. Condensins depletion is known to perturb structure and function of centromeres, however the mechanism of this functional link remains elusive. Depletion of condensin activity is now shown to result in a significant loss of loading of CENP-A, the histone H3 variant found at active centromeres and the proposed epigenetic mark of centromere identity. Absence of condensins and/or CENP-A insufficiency produced a specific kinetochore defect, such that a functional mitotic checkpoint cannot prevent chromosome missegregation resulting from improper attachment of sister kinetochores to spindle microtubules. Spindle microtubule-dependent deformation of both inner kinetochores and the HEC1/Ndc80 microtubule-capturing module, then results in kinetochore separation from the Aurora B pool and ensuing reduced kinase activity at centromeres. Moreover, recovery from mitosis-inhibition by monastrol revealed a high incidence of merotelic attachment that was nearly identical with condensin depletion, Aurora B inactivation, or both, indicating that the Aurora B dysfunction is the key defect leading to chromosome missegregation in condensin-depleted cells. Thus, beyond a requirement for global chromosome condensation, condensins play a pivotal role in centromere assembly, proper spatial positioning of microtubule-capturing modules and positioning complexes of the inner centromere versus kinetochore plates.

## Introduction

The establishment and maintenance of chromosome condensation are essential cell cycle steps that facilitate accurate chromosome segregation and therefore ensure the integrity of eukaryotic genomes. Condensin complexes play a key role in chromosome condensation and are essential for chromosome segregation [1]. Fission and budding yeast have only one condensin complex (condensin I) [2], [3], while multicellular organisms usually have two: I and II [4], [5]. These two condensin complexes both consist of five subunits: two shared SMC subunits SMC2/CAP-E and SMC4/CAP-C, which form the enzymatic (ATPase) and structural core of the complex, and three complex-specific non-SMC subunits: CAP-D2, CAP-G, CAP-H (condensin I) and CAP-D3, CAP-G2, CAP-H2 (condensin II). The two condensins have different timing of loading onto chromatin and form distinct patterns of enrichment along each condensed chromosome [6], [7], [8]. *In vitro* biochemical analysis has uncovered that purified condensin I can reshape a bound DNA molecule in an ATP-dependent manner [9].

One prominent aspect of condensins' activity *in vivo* is a specific function at centromeres. The role of condensins in maintaining proper centromere structure has been reported in many metazoan systems and, recently, in yeast. Despite a higher enrichment of condensin II in centromeric chromatin (near the inner kinetochore plate), condensin I apparently plays a bigger role in correct sister centromere separation [6]. Nevertheless, condensin I and II appear to cooperate in facilitating the proper segregation of sister centromeres in anaphase [10], [11]. Condensins' localization largely overlaps with CENP-A, the centromeric histone H3, but appears to be internal to kinetochores themselves [6], [12]. Furthermore, condensin II likely requires CENP-A for recruitment to the centromere [12]. Both condensin complexes are also regulated (apparently indirectly) by Aurora B, specifically at the centromeres [6], [8], [13], [14]. In particular, the early step of condensin I binding to centromeres is Aurora B dependent, while the role of Aurora B in condensin II loading is controversial: [6] versus [8]. It is also unknown whether Aurora B functions after kinetochore assembly involves regulation of condensins.

The actual contribution of condensins to centromere/kinetochore function is not understood. In yeast condensin mutants, sister centromeres are able to orient properly to reach metaphase [3], [15], and small centromere-containing minichromosomes segregate with high fidelity [16], [17]. At the same time, a recent study in budding yeast that surveyed the localization of key centromere and kinetochore complexes in condensin mutants [18] has revealed that Cse4p (CENP-A) localization is severely disrupted. This disruption apparently induces centromere stretching and continued activation of the mitotic checkpoint (also known as the spindle assembly checkpoint) [18], which enables prolonged pre-anaphase arrest, thereby allowing for proper bipolar attachments of sister kinetochores [19]. However, metazoan sister centromeres cannot separate properly upon condensin inactivation, especially in the absence of condensin I, and the structure of centromeric chromatin becomes disorganized and stretched, so that kinetochores are frequently seen far outside the chromosome mass [6], [20]. Nevertheless, in condensin-depleted metazoan cells, targeting of centromere and kinetochore markers is largely normal [6], [21]. Condensin-dependent loss of CENP-A has not been reported in vertebrates, and even the ability of condensin defects to trigger the mitotic checkpoint [20] has been questioned [22]. Unlike yeast mutants, condensin-depleted cells of higher eukaryotes enter anaphase with transient delay [7], but with prominent centromere defects [20] and uncoordinated chromosome movement [6], [23] that result in chromosomal bridges arising from missegregated centromeres [6].

Here, we have investigated the nature of condensin function in human centromeres, including structure of both inner and outer kinetochores, microtubule attachment, and mitotic checkpoint activation and silencing. Our findings indicate that condensins function to prevent centromere defects, which likely originate and/or are accompanied with depletion of CENP-A and a loss of centromere rigidity in mitosis. When condensins are depleted, the resulting dispersed microtubule attachment sites mediate massive merotelic attachments. Furthermore, Aurora B activity at centromeres, which is needed to correct improper microtubule attachments, is partially reduced upon condensin dysfunction. Therefore, in condensin-depleted cells, despite the functioning kinetochore-dependent mitotic checkpoint, chromosomes segregate with improperly attached kinetochores, resulting in breaks within chromosome bridges that occur in late anaphase. Thus, condensin dysfunction synergistically induces merotelic attachments and compromises the merotelic attachment correction mechanism, leading to chromosome breaks and nondisjunction.

## Results

### Mitotic checkpoint activation without condensin

**Figure 1:**
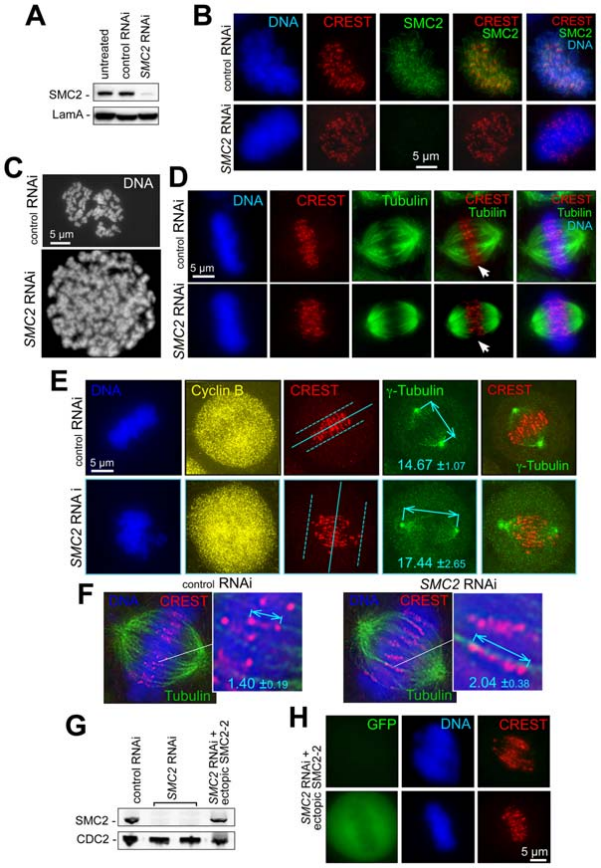
Depletion of condensins 1 and 2 disorganizes metaphase chromosome alignment and centromere structure. A) SMC2 depletion by RNAi. Immunoblotting with specific condensin antibodies (see Methods) four days after siRNA transfection; Laminin A – loading control. Indirect immunofluorescence images of fixed preparations of HeLa cells transfected with *SMC2* siRNA or mismatched siRNA (*control RNAi*) and stained with DAPI (DNA) and anti-SMC2 antibodies four days after transfection. B) Disorganization of metaphase alignment in condensin-depleted cells. Fixed preparations of HeLa cells transfected with *SMC2* siRNA or mismatched siRNA (*control RNAi*) were stained with DAPI (DNA), anti-centromere (CREST) and anti-SMC2 antibodies 4 days after transfection. SMC2 depletion always produced a diffuse metaphase plate, accompanied by the loss of visual separation between sister centromeres and their delocalization from the spindle mid-zone (Fig. 1D–1F). C) Chromosome condensation defects in SMC2-depleted cells. Chromosome morphology was analyzed by spreading on the fourth day after transfection with SCM2 siRNA. Cells were treated with demecoline for four hours and mitotic cells were collected by shake-off. Chromosome spreads were prepared as described in Methods. D) Disorganization of metaphase alignment in condensin-depleted cells. Loss of visual separation between sister centromeres (CREST staining) and their delocalization from the spindle mid-zone after SMC2 depletion. Only partial Z-section projections are shown in CREST/tubulin panels (marked with arrows) to better illustrate the loss of ordered metaphase alignment. E) SMC2 depletion causes elongated spindles and reduced inter-polar tension. HeLa cells were treated as in (A) and stained with anti-cyclin B (cell cycle marker), anti-γ-tubulin (spindle pole marker) and CREST antibodies. DNA is stained with Hoechst 33342. Deconvolved images were used for spindle length measurements (shown with arrows) and spindle midzone positioning (solid line at CREST panel) using the anti-γ-tubulin label. Dashed lines denote the dispersion of centromeres in condensins-depleted cells versus control. F) SMC2 depletion produces elongated and deformed centromeres. HeLa cells treated as in (E) are stained with α-tubulin antibodies (spindle marker), CREST antibodies and Hoechst 33342. The distances between the outer edges of sister centromeres were measured in deconvolved (as shown) images. G,H) Ectopic expression of RNAi-resistant SMC2-2 restores SMC-2 levels, chromosome alignment, and centromere stretching. G) Immunoblotting for endogenous SMC2 or ectopic SMC2-2 mutant protein detected four days after siRNA transfection and two days after SMC2-2 transfection. CDC2 – loading control. H) HeLa cells transfected with *SMC2* siRNA or mismatched siRNA (*control RNAi*) were retransfected 48 h later with a mixture of SMC2-2- and GFP-expressing plasmids. More than 95% of GFP-positive metaphases had wild-type metaphase plate morphology (bottom panels), while GFP-negative cells (upper panels) showed characteristic condensin-depletion defects.

Condensin-depleted metazoan cells enter anaphase accompanied by chromosome missegregation [11], [20], [24], [25], an outcome in apparent contrast with yeast conditional condensin mutants, where mitotic checkpoint activation protects chromosomes from missegregation and is essential for recovery after cells return to permissive conditions [18]. To address this apparent difference, mitotic checkpoint signaling within human cells was examined after depletion of condensins. As condensin I and II appear to have an additive effect on centromere structure ([6] and Fig. S1), all subsequent experiments in human cells were conducted with *SMC2* RNAi, which inactivates both condensin I and II (Fig. 1A–D). Both disorganized metaphase (Fig. 1B, D) and centromere stretching (detected with CREST antibody; staining includes the inner kinetochore and chromatin connecting the two sisters) (Fig. 1E, F) were associated with SMC2 depletion, as expected. Pole-to-pole spindle length and the distance between the leading edges of the CREST signal in SMC2-depleted cells were both increased by 20% and 45%, respectively (Fig. 1E, F). Both metaphase and centromere disorganization were condensin-loss specific, as a control mismatch siRNA did not show these defects, and complementation of these defects was demonstrated upon double transfection of *SMC2* siRNA and a construct expressing a mutant siRNA-resistant SMC2-2 protein (containing a mismatch mutation at the siRNA site, which does not change encoded aminoacid sequence) (Fig. 1G, H).

**Figure 2:**
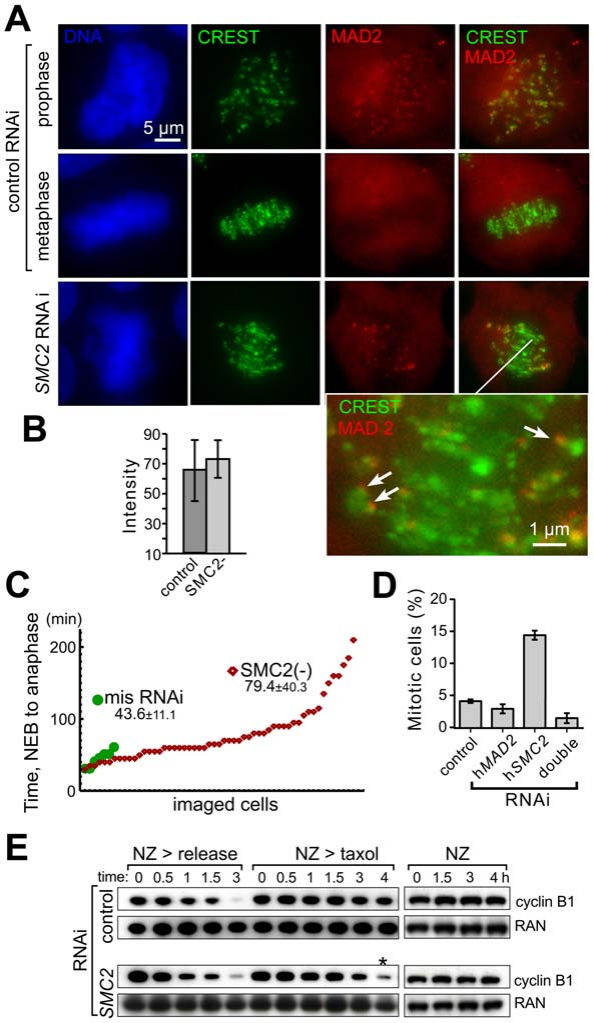
Condensin depletion activates the mitotic checkpoint but compromises its maintenance. A) hSMC2 depletion results in a prometaphase-like delay with proper MAD2 localization to unattached kinetochores. Asynchronous HeLa cells were transfected with either specific *SMC2* siRNA (*SMC2* RNAi) or the control mismatch siRNA. The fixed cells are stained with anti-hMAD2 and anti-centromere CREST antibodies, DNA - with Hoechst 33342. Higher magnification detail for the lower right panel shows examples of unattached (MAD2-containing) kinetochores (arrows) in an SMC2-depleted cell. B) MAD2 intensity at unattached kinetochores is at wild type level in SMC2-depleted cells. MAD2 signal fluorescent intensity was measured in prophase control cells and prometaphase condensin-depleted cells using MetaMorph software (Molecular Devices Corp., Downington, PA). Average intensity for at least 50 individual MAD2-stained kinetochores per experiment was measured using same-size regions of interest encircling the MAD2 signals. C) Anaphase is delayed is induced in condensin-depleted cells. Mitotic progression in control (control RNAi) and condensin-depleted (SMC2-) cells. Mitotic progression in individual cells was monitored with time-lapse microscopy (5-min intervals) of HeLa cells stably expressing H2B-GFP [61]. The chromatin/chromosome morphology was used to determine the timing from nuclear envelope breakdown (NEB) to the onset of segregation. Timing determined for individual cells (arranged from shortest to longest time). D) hMAD2 depletion eliminates mitotic delay in SMC2-depleted cells. The mitotic index was determined by scoring cell morphology in live cultures 3 days after siRNA transfection to reduce SMC2. E)) Condensin-deficient cells are proficient in the maintenance of the mitotic checkpoint in the absence of kinetochore tension. Immunoblotting pf cell extracts to determine the kinetics of cyclin B1 levels in nocodazole-arrested cells following release into fresh media (*NZ>release*), release into taxol-containing media (*NZ>taxol*), and kept in nocodazole for 4 h (*NZ*). RAN levels are shown as loading control. The asterisk denotes cyclin B degradation in taxol-released condensin-depleted cells.

Such a profound centromere and metaphase disorganization did not appear to affect functionality of the mitotic checkpoint. Indeed, MAD2 was found to recognize and bind to individual unattached kinetochores in condensin-depleted prometaphase cells (Fig. 2A) essentially at the wild-type prophase levels (Fig. 2B). This indicates that condensins-depleted cells have normal level of MAD2 response but delayed timing of full kinetochore attachment. No MAD2 staining was detected at kinetochores in anaphase cells (data not shown). Time-lapse microscopy of live cells cycling in the absence of microtubule drugs revealed that condensin depletion induced a substantial prometaphase/metaphase delay (Fig. 2C) that tripled the mitotic index in a MAD2-dependent manner (Fig. 2D), consistent with prior reports [7], [26]. When all kinetochores were unattached after microtubule disassembly induced with nocodazole, condensin-depleted cells were as proficient as wild type cells in maintaining a sustained mitotic checkpoint arrest (indicated by the stabilization of the B1 cyclin, Fig. 2E). However, after taxol treatment, which lowers intrakinetochore stretch (and tension development) after attachment of sister kinetochores [27], mitotic checkpoint signaling was not sustained in condensin-depleted cells, resulting in cyclin B1 degradation (Fig. 2E) and anaphase entry. Thus, defects in condensin-depleted kinetochores induce longer mitotic-checkpoint-dependent delay, apparently slower attachment to spindle microtubules, but accompanied by reduced ability to maintain checkpoint signaling.

As a result, the putative structural defects in condensin-depleted chromosomes persist through anaphase, where SMC2-depleted cells fail to segregate a substantial proportion of chromosomes. Later, in telophase, this nondisjunction is apparently resolved via putative chromosomal breaks, which were identified by appearance of the foci of phosphorylated histone H2AX (Fig. S2). Thus, the most reasonable view is that both reduced ability to maintain mitotic checkpoint and the ensuing chromosome bridges and breaks in condensins-depleted human cells are dependent on kinetochore dysfunction stemming from improperly structured centromeric chromatin.

### Centromere damage from reduced loading of CENP-A without condensin

**Figure 3:**
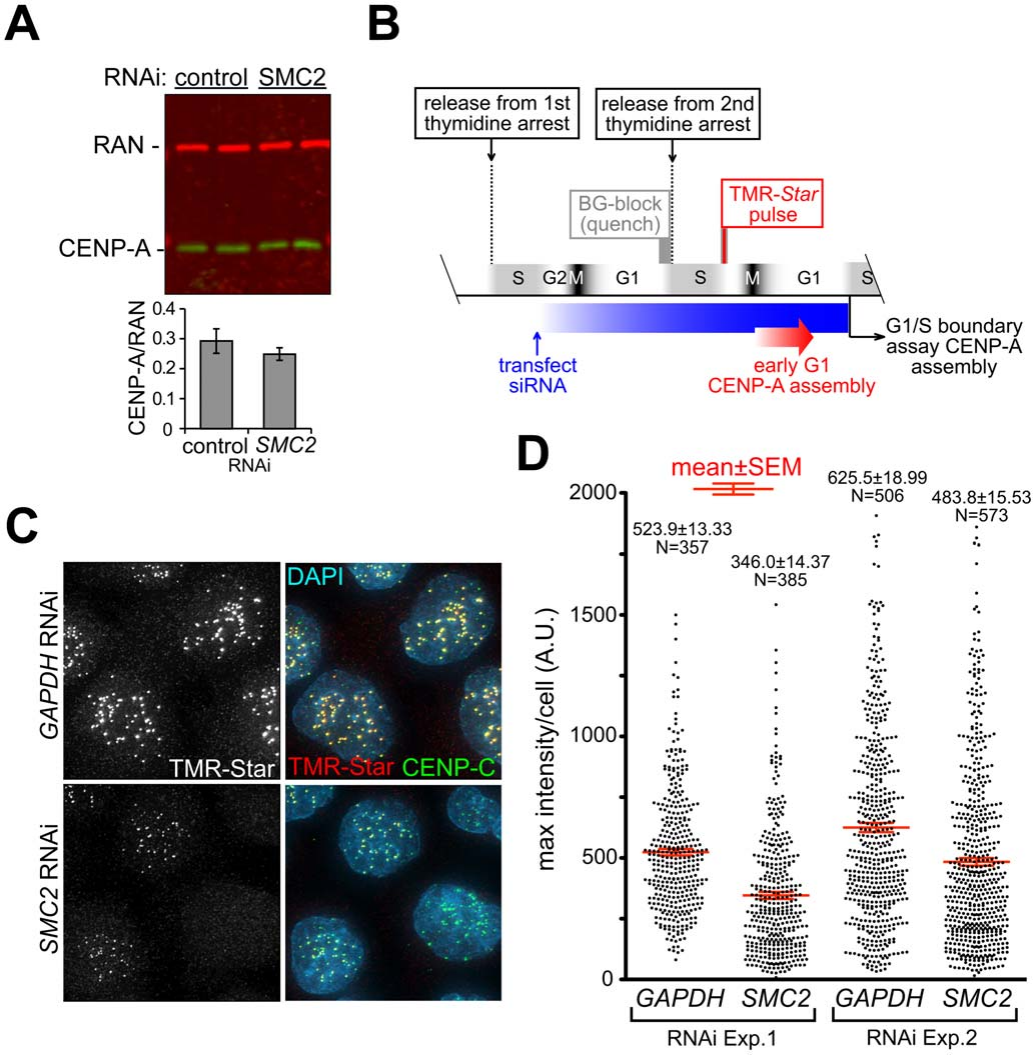
Efficient assembly of CENP-A at centromeres requires SMC2. A) CENP-A levels are not significantly decreased in condensin-depleted cells. CENP-A levels were assessed in two independent samples of control and SMC2-depleted cells (both nocodazole-arrested as in Fig. 2E, 3 hr timepoint). Immunoblotting for CENP-A and RAN (as a loading control), quantified with Li-Cor Odyssey scanner using alternative fluorescent dyes. B) Schematic of cell synchronization, RNAi and SNAP-labeling strategy. CENP-A-SNAP synthesized during S phase was specifically fluorescently labeled in late S/G2 and the accumulation at centromeres was measured after mitosis at the end of the next G1. C) Results of procedure in (B). CENP-SNAP assembly at centromeres following indicated RNAi shown by TMR-*Star* labeling. Centromeres are counterstained with anti-CENP-C. D) Fluorescent intensity distribution of peak TMR-*Star* signal per cell from two independent experiments. The means and the standard errors of the mean (SEM) are shown for each experiment. The differences between the control RNAi and condensin depletion were statistically significant (one-tailed P-values<0.0001 in T-test with significantly different variances; t = 9.04 df = 740 in exp.1, t = 5.83 df = 1077 in exp.2). Eleven data points above 2000 were counted but are not shown on the graph.

The most probable interface between condensin activity and kinetochore functions is centromeric chromatin. Condensin has been reported in non-mammalian systems to interact both physically [28] and functionally [13], [18] with the centromere specific histone H3 variant CENP-A. We have reported that in yeast CENP-A (Cse4) was notably depleted from centromeres in the absence of active condensin [18]. In contrast, condensin depletion from HeLa cells left overall CENP-A levels unchanged (Fig. 3A). However, since CENP-A has a complex loading dynamics, with its stable assembly into centromeric chromatin restricted to the earliest portion of the G1 cell cycle phase, a condensin contribution to loading might have been masked by the bulk pool of this protein [29].

To test if centromeric chromatin was affected by condensin depletion in human cells, we used a pulse-labeling technique to visualize the assembly of new CENP-A into centromeric chromatin after condensin loss with *SMC2* siRNA (Fig. 3B). For this, we exploited the SNAP tag, which consists of a 20 kD suicide enzyme that has been mutated to use benzylguanine as a substrate [29]. HeLa cells stably expressing SNAP-tagged CENP-A were synchronized at the G1/S boundary or within S phase, transfected with siRNA to condensin after release into S phase, and arrested at the subsequent G1/S boundary by addition of thymidine. Existing SNAP-CENP-A (loaded into chromatin and the chromatin-free pool) was reacted to completion with non-fluorescent benzylguanine. The cells were released again into S phase and TMR-Star (rhodamine-labeled benzylguanine) was added to covalently label the newly synthesized SNAP-CENP-A (Fig. 3B). Assembly of CENP-A at centromeres was assessed after collecting cells at the subsequent G1/S boundary (again by addition of thymidine) and quantified by fluorescent microscopy (Fig. 3C). As seen before [29], CENP-A loading was restricted to late telophase or early G1 (not shown). A reproducible two-fold decrease in CENP-A centromere loading (relative to cells with normal condensin level) was seen in condensin-depleted cells (Fig. 3D). Thus, human cells are unable to maintain CENP-A levels at centromeres upon condensin loss, demonstrating a novel condensin-dependent centromere defect in vertebrates.

### Aberrant kinetochore structure and function without condensin

**Figure 4:**
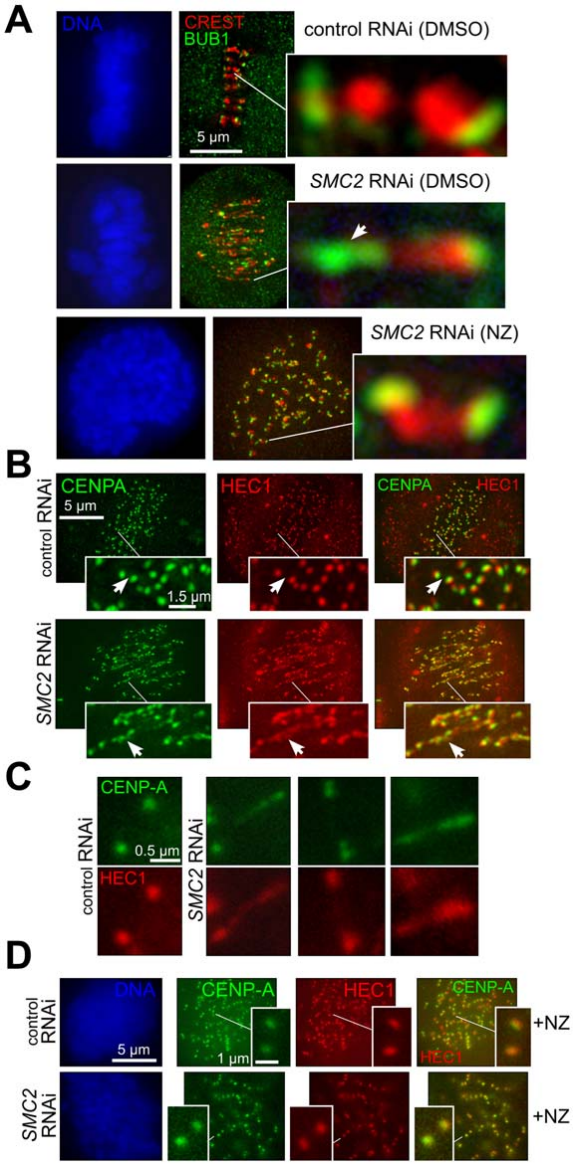
Both inner and outer kinetochore proteins are stretched/dispersed by spindle forces in metaphase upon SMC2 depletion. A) Abnormal stretching of inner kinetochores in condensin-depleted chromosomes depends on spindle forces. HeLa cells treated as in (Fig. 2A) were additionally exposed to nocodazole (NZ) or DMSO for 16 hours and stained with anti hBUB1 and CREST antibodies. Deconvolved images were used to score individual sister centromere/kinetochore pairs (inserts), which due to centromere stretching would be impossible to identify in condensin-depleted cells without such a double staining. B) Microtubule-anchoring HEC1/NDC80 complex is stretched upon SMC2 inactivation. Asynchronous HeLa cells were treated as in Fig. 2A and stained with ant-HEC1 (labeling the microtubule-interacting HEC1/NDC80 complex) and anti-CENPA (centromere chromatin marker) antibodies. C) Examples of different degrees of putative merotelic attachments based on the morphology of outer kinetochore in SMC2-depleted cells. Cells were treated and stained as in (B). D) The spindle-generated tension contributes to HEC1 dispersion in condensin-depleted kinetochores. Cells treated as in (A) were additionally incubated with nocodazole (+NZ) for 16 hours prior to fixation and staining.

While centromeric chromatin deformation and stretching in the absence of condensins (Fig. 1) has been described in several metazoan systems [6], [7], [30], the nature of kinetochore defects [6], [23] has not been elucidated, and the existence of such defect itself has been called into question by other investigators [20], [31]. SMC2-depleted HeLa cells stained with antibodies against BUB1 to visualize the inner kinetochore plate [32] showed abnormally stretched BUB1 localization, with sister kinetochore pairs located outside of the central zone and frequently displaying only one kinetochore visibly stretched, a pattern consistent with merotelic attachment (Fig. 4A). Importantly, stretching of both signals for CREST and BUB1 was microtubule-dependent, as it was not seen when microtubule assembly was blocked (Fig. 4A). A similarly stretched morphology of the inner kinetochore (visualized by *Xenopus* BUB1 antibody) was observed in *Xenopus laevis* sperm chromosomes *in vitro*, upon depletion of xSMC2 (data not shown), indicating that the observed kinetochore deformations are not unique to cultured human cells.

Positioning of the outer kinetochore component NDC80/HEC1, which makes direct contact with kinetochore microtubules [33], [34], [35], [36], was also abnormally elongated in HeLa cells upon SMC2 depletion (Fig. 4B). A survey of individual sister kinetochore pairs revealed a broad range of severity from mild to severe in HEC1 dislocation, sometimes differentially stretching one sister kinetochore (Fig. 4C). This observation and the fact that both BUB1 and HEC1 stretching are microtubule-dependent (Fig. 4A,D) may indicate that the kinetochore deformation is at least partially due to microtubule mis-attachments.

### Merotelic microtubule attachments without condensin

**Figure 5:**
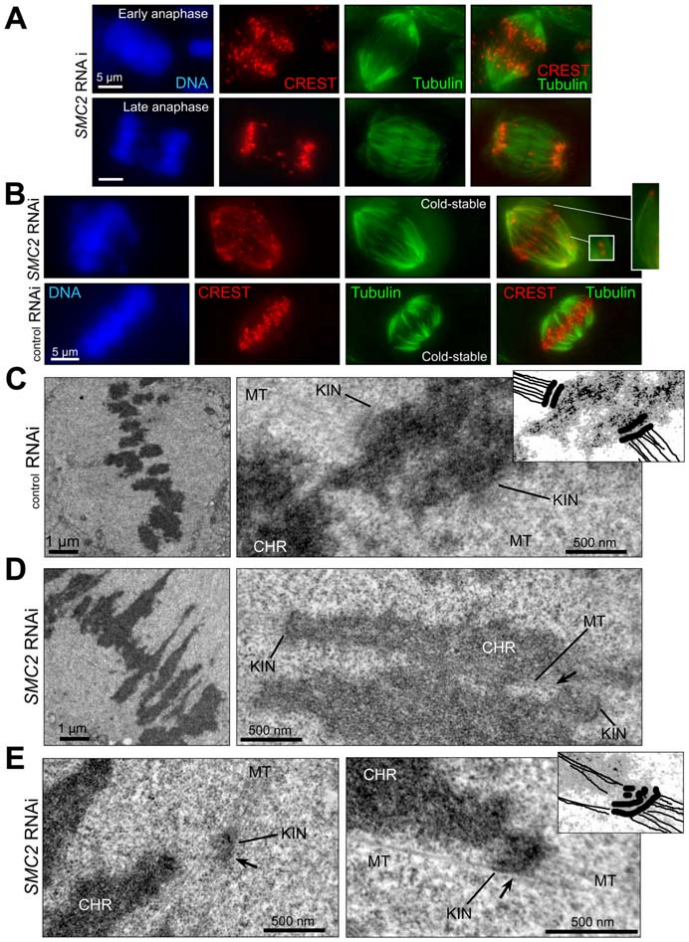
Kinetochore defects in condensin-depleted cells lead to a high incidence of syntelic and merotelic mis-attachments of kinetochore microtubules, which are not corrected prior to anaphase onset. A) Condensin-less anaphase has an abnormally high frequency of lagging chromosomes. An example of anaphases in condensin-depleted HeLa cells with centromeres labeled with CREST antibodies and spindle microtubules with anti-α-tubulin antibodies. The estimated frequency of lagging and/or non-disjoined centromeres is more than 20%, as compared to less than 1% in wild type. The lagging centromeres were defined as falling outside the distance to the pole for the bulk of centromeres in untreated cells. As many centromeres were extremely stretched, and were not counted as a result, the actual lagging number should be substantially higher. B) A high incidence of syntelic mis-attachments of kinetochore microtubules in metaphase upon condensin depletion. HeLa cells were treated as in Fig. 2A, except that they were incubated on ice for 10 min prior to fixation and staining as in (A). The magnified region shows syntelic mis-attachment of individual chromosomes. Many attachments appear to be merotelic, as indicated by centromere stretching. C) Normal bipolar attachment of sister kinetochores to microtubules in condensin-proficient HeLa cells. HeLa cells treated with mismatched siRNA (mis RNAi) were processed for and imaged by transmissive electron microscopy (see Methods). Microtubules (MT) interact with sister kinetochores (KIN) from opposite poles in these control cells. No incidence of merotelic attachments or stretched centromere chromatin was detected (more than 100 kinetochores imaged). The insert schematic depicts the arrangement of sister kinetochores with attached microtubules. Lower magnification (left) shows the entire metaphase plate. D) Condensin depletion results in stretched centromeric chromatin and deformed kinetochore layers, possibly caused by merotelic mis-attachments. An example of two neighboring kinetochores in SMC2-depleted cells (*SMC2* RNAi) is shown (right panel). The extreme stretching of centromere chromatin is accompanied by a deformed and rounded kinetochore structure, which possibly indicates some degree of merotelic attachments to both kinetochores. Lower magnification (left panel) shows the typical view of metaphase in condensin-less cells. E) Merotelic mis-attachment is pervasive in condensin-depleted human cells. Two examples of moderate merotelic mis-attachments of kinetochores in hSMC2-depleted cells are shown (right panel). At least half (arrows) of the kinetochore in each case is attached to the opposite pole. The insert schematic shows the arrangement of a merotelic kinetochore with attached microtubules. It is likely only a small fraction of merotelic kinetochores are identifiable in each metaphase, as most are extremely stretched (i.e. probably double-merotelic) based on light microscopy and immuno-EM data (Fig. S3).

Microtubule-dependent kinetochore stretching after depletion of condensin would be consistent with bi-oriented attachment and subsequent stretching of a more “elastic” centromere/kinetochore in the absence of condensin. In this case one would observe a clearly separate pair of stretched kinetochores, which rarely observed in condensin-depleted cells. Alternatively, kinetochore stretching might be caused by merotelic attachment, in which microtubules from both spindle poles attach to a single kinetochore. A high degree of merotely on both sister kinetochores would potentially create sister pairs where both centromere and kinetochore are stretched resulting in visually irresolvable pairs, as frequently observed upon condensin depletion (Fig. 4B). Most merotelic attachments in wild type affect only one sister kinetochore and are corrected prior to anaphase, any (few) remaining mis-attachments are resolved upon application of anaphase pulling forces [37]. Double staining with kinetochore and centromere markers identified some merotelic pairs after condensin depletion (Figs. 4A, 4C). Direct probing of every kinetochore pair for merotelic attachments is technically unachievable: there are no reliable biochemical markers specific for merotelic kinetochores in mammalian cells, and the resolution level of light microscopy does not enable robust imaging of all microtubules attached to a single kinetochore. However, a surrogate measure for unresolved merotelic attachment by the time of anaphase initiation is the retarded anaphase movement of those affected kinetochores. In SMC2-depleted anaphases, kinetochores appeared to move discordantly with each other (Fig. 5A), consistent with massive merotelic attachment. Furthermore, reflecting the high propensity of condensin-depleted kinetochores to misattachment, some chromosomes were apparently attached syntelically, i.e. with both sister kinetochores attached to the same pole (Fig. 5B, insert).

Electron microscopy (EM) was used to investigate at higher resolution the extent of merotelic mis-attachments in condensin-deficient kinetochores. In wild type cells with normal condensin activity (Fig. 5C), kinetochores formed a flat tri-laminar structure, with sister kinetochores separated by only about 1 µm. In each instance, both sister kinetochores were found in close proximity; no merotelic attachments were identified. In condensin-depleted cells, the deficits in kinetochore assembly (especially stretching) made most kinetochores more difficult to identify morphologically (Fig. S3). For those that could be identified in condensin-depleted cells, kinetochore layers were usually bent and sister kinetochores were never found close to each other (Fig. 5D). Moreover, upon condensin depletion, more than one-third of identifiable kinetochores showed morphology and microtubule positioning consistent with attachment to both poles, resulting in the kinetochore surface being bent backwards at a significant angle (Fig. 5E). The EM images (Fig. 5) collectively reinforce the correlation between stretching of centromeric chromatin, spreading of microtubule-binding modules of individual kinetochores and a net increase in uncorrected syntelic, merotelic, and double-merotelic attachments in condensin-depleted cells.

### Disregulated Aurora B after condensin depletion

**Figure 6:**
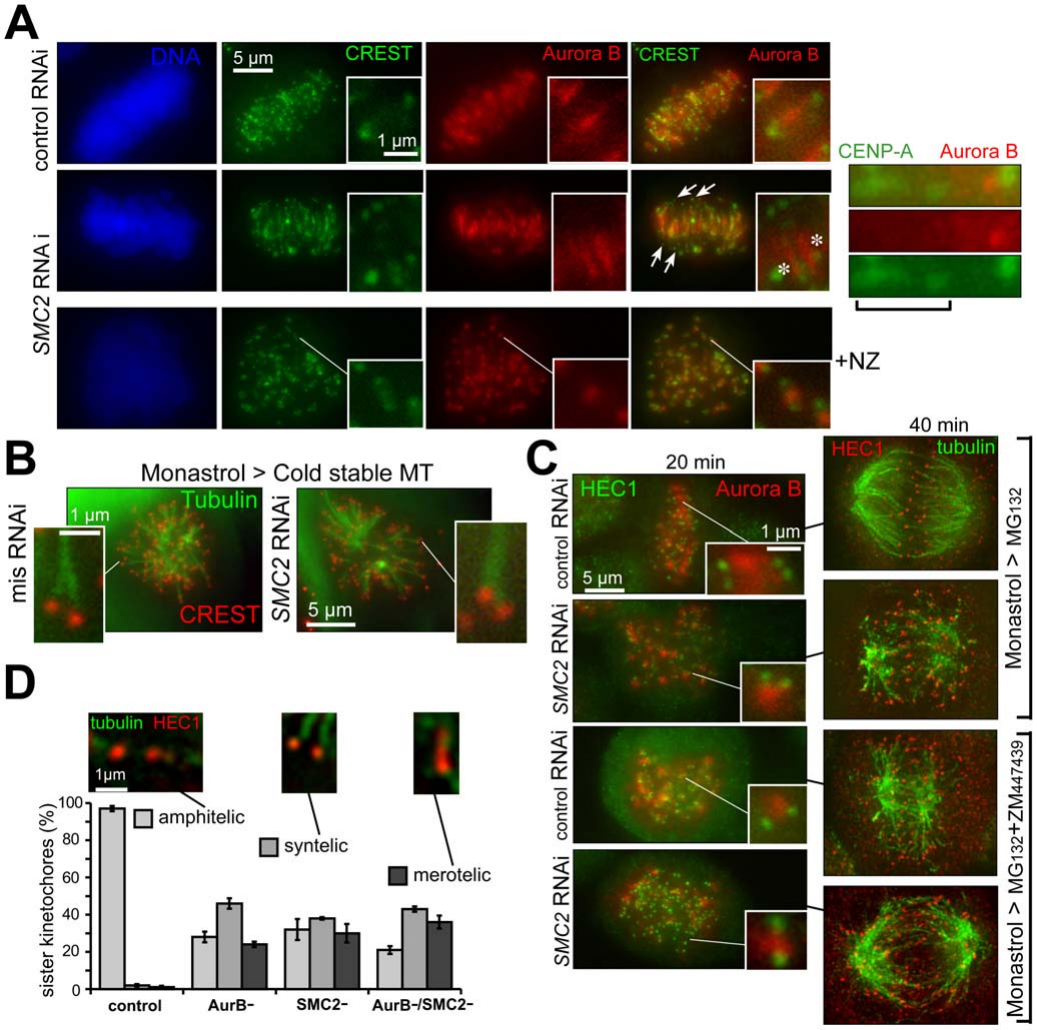
Condensin depletion mimics the disruption of Aurora B activity in correcting kinetochore microtubule misattachments. A) Two modes of Aurora B mislocalization between condensin-depleted sister centromeres. Left panels: Localization of Aurora B kinase (anti-Aurora B antibodies) is shown relative to kinetochores (CREST sera) in control cells (mis RNAi), condensins-depleted cells (*SMC2* RNAi) and in condensin-depleted cells treated by nocodazole (NZ). Significant separation between CREST and the bulk of Aurora B signals is evident in *SMC2* RNAi cells. Inserts show magnified images representing individual sister kinetochore pairs from single optical sections. The arrows point to the Aurora B – free centromeres, which made a long excursion away from metaphase plate; asterisks indicate abnormally asymmetrical localization of Aurora B between condensin-depleted sister centromeres. Right panel: an example of preferentially reduced Aurora B staining at the stretched (highly merotelic) centromeres (indicated by brackets) within a sister pair. B) Monastrol arrest produces syntelic chromosomes in SMC2-depleted cells. Monastrol-treated cells, SMC2-depleted or control, were also treated with cold to stabilize microtubules. C) SMC2 depletion and Aurora B inactivation have similarly uncorrected merotelic metaphases. Analysis of cells released from monastrol arrest in the presence of condensins depletion, Aurora B inactivation (by ZM447439) or both showed that more than 90% of metaphases remain uncorrected after release (disorganized with high proportion of misattached microtubules), versus only 10% in control cells. Staining for HEC1 and Aurora B is shown for 20 min after release, HEC1 and microtubules – 40 min. D) SMC2 depletion and Aurora B inactivation have similar levels of abnormally attached kinetochores. Around 200 kinetochores (in cells stained for HEC1 and α-tubulin) were counted for each experiment 40 min after release from monastrol. Examples of qualifying images are shown for each category, determined on the basis of kinetochore position, stretching, and microtubule configurations. Statistical analysis confirmed that distribution of attachment types is indistinguishable between the condensins depletion, Aurora B inhibition and double treatment (Chi-square test: amphitelic 0.54 p>0.75, syntelic 0.35 p>0.85, merotelic 0.21 p>0.9).

While it is plausible that the stretched kinetochores in condensin-depleted chromatin have disoriented microtubule-binding sites that facilitate merotelic attachments, why would such mis-attachments remain uncorrected in metaphase and anaphase? Our data, as well as the reported sensitivity of INCENP localization to condensin function [20], suggest a possibility that Aurora B, which is known to facilitate detachment, severing and reattachment of microtubules from the incorrect pole [35], [38], [39], [40], is itself mislocalized and/or inactivated upon depletion of the condensins. Immunofluorescent staining for Aurora B revealed that while this kinase is still present between sister kinetochores of condensin depleted cells, its positioning is both stretched in a microtubule-dependent manner and positioned asymmetrically between sister centromeres (Fig. 6A, asterisks). As a result, up to 50% of centromeres, which separate far from the midzone, have no visible Aurora B, and thus merotelic errors in their attachment cannot be corrected (Fig. 6A, arrows). Furthermore, in the few identifiable asymmetric sister centromere pairs, where one sister centromere is more prominently stretched, we found that the latter has a decreased level of Aurora B (e.g., Fig. 6A, right panel).

To test if Aurora B activity was compromised by condensin depletion, we initially used the Eg5 inhibitor monastrol [41] to induce syntelic kinetochore attachments that are easily identifiable by light microscopy. Like merotelic attachments, syntelic attachments are corrected by the same Aurora B dependent pathway [39], [42]. During recovery of either control or condensin-depleted cells from treatment with monastrol (Fig. 6B), cells were treated with proteasome inhibitor MG132, resulting in pre-anaphase arrest. Cell populations as a whole, as well as individual pairs of sister kinetochores, were assayed for recovery of bipolar attachment (Fig. 6C). The effect of inhibition of Aurora kinase activity was determined by treatment with a selective Aurora inhibitor (ZM447439). For the cell population analysis, both condensin depletion and Aurora B inhibition showed a similar (90%) proportion of abnormal (unrecovered) metaphases with ample signs of syntelic and merotelic attachments still persisting as late as 40 min after the release from monastrol. Moreover, both condensin depletion and Aurora B inhibition appeared to be epistatic, each yielding the same 90% of unrecovered (disorganized and/or merotelic) metaphases. Analysis of individual pairs of sister kinetochores confirmed that depletion of condensin appears to phenocopy (Fig. 6D) Aurora B inhibition. The persistence of unrecovered syntelic attachments also indicate that there is apparently a constitutive defect of Aurora B activity at centromeres upon condensin depletion, in addition to the tension-specific defect (as suggested by Fig. 6A).

**Figure 7:**
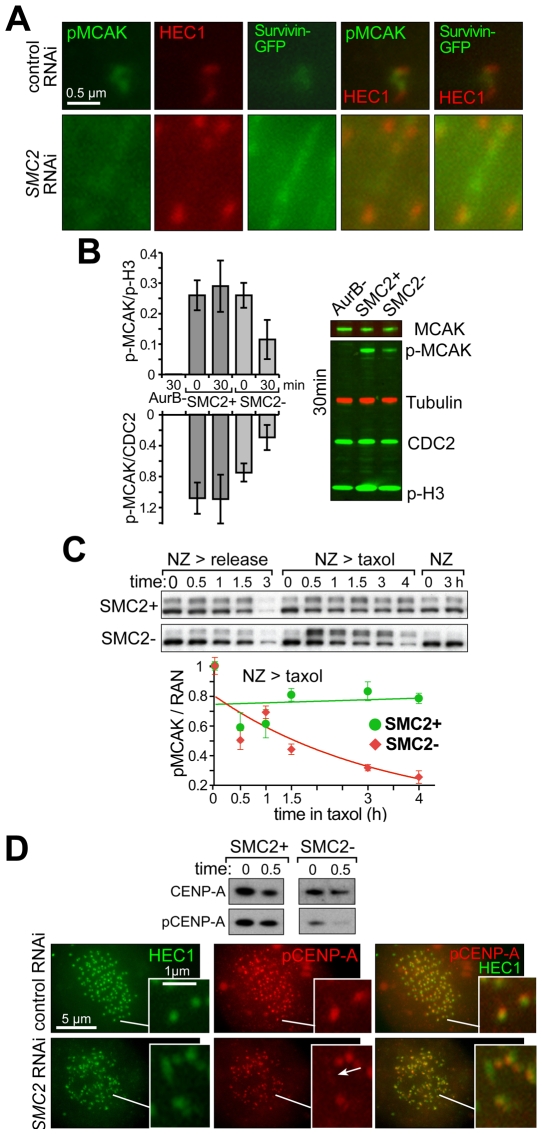
Condensins depletion decreases Aurora B activity at kinetochores. A) Merotelic mis-attachment of kinetochores in SMC2-depleted cells reveals reduced MCAK phosphorylation. HeLa cells stably expressing Survivin-GFP [32] were stained by HEC1 and phospho-S95 MCAK antibodies. B) Centromere-specific phosphorylation of MCAK at S95 is reduced in condensin-depleted cells as a result of spindle force application. Total HeLa extracts (0 and 30 minutes after release from monastrol arrest) were analyzed for the level of phosphorylation of MCAK using phospho-S95 antibodies in the presence of Aurora B inhibitor (ZM44743), in control cells and condensins-depleted cells. Anti-MCAK, α-Tubulin, CDC2 (anti-PSTAIRE antibody) or phospho-H3 (p-S10) was used as loading controls. Immunoblotting signals (only 30 min are shown) were quantified with a Li-Cor Odyssey scanner. Phospho-MCAK, phospho H3 and α-tubulin were always detected in the same gel (membrane), using alternative fluorescent dyes. The absolute decrease of MCAK phosphorylation was reproducibly detected upon Aurora B inhibition and condensin depletion (gel image). The relative decrease was also evident relatively to loading controls: MCAK (determined from a separate gel), CDC2 and tubulin. The graph shows S95 phospho-MCAK level normalized either to the phospho-S10 H3 signal, to isolate the centromere-specific component of Aurora B activity, or to CDC2 to illustrate the absolute decrease of MCAK phosphorylation. Two time points, 0 and 30 min, are shown. Standard deviations result from at least three independent experiments. C) Condensins-deficient cells cannot maintain proper level of active Aurora B at kinetochores in the presence of kinetochore tension. Immunoblotting reveals the kinetics of phospho-S95-MCAK levels (all thee bands are S95-phosphorylated MCAK forms) in purified chromatin of nocodazole-arrested cells released into fresh media (*NZ>release*), released into taxol-containing media (*NZ>taxol*), and kept in nocodazole for 3 h (*NZ*) (procedure as in 2E). RAN levels in total extracts (not shown) were used for phospho-MCAK signal normalization (graph) using a Li-Cor Odyssey scanner on the basis of two experiments. D) Phosphorylation of CENP-A at Ser7 is reduced in condensin-depleted cells. Immunoblotting - used to determine the level of phosphorylation of CENP-A in purified chromatin (0 and 30 min after release from nocodazole arrest into fresh media) with the specific anti-phospho-CENP-A (p-Ser7) antibody. Total CENP-A levels are shown as loading controls. A reproducible phospho-CENP-A decrease was found at both time points in three independent experiments. The micrograph shows fixed asynchronous HeLa cells that were stained with HEC1 and phospho-CENP-A antibodies. The insert highlights the additional reduction of CENP-A phosphorylation at the sister centromere that is more stretched (merotelic) in hSMC2-depleted cells.

Inhibition of Aurora B at centromeres would impair the HEC1 and MCAK - mediated [36], [40], [43], [44], [45] pathway of correcting merotelic kinetochores. To directly assess Aurora B enzymatic activity at centromeres, we first followed phosphorylation of hMCAK/KIF2C at position S95 (in the human protein), a modification that participates in regulating MCAK localization to centromeres [44]. Simultaneous monitoring the positions of MCAK (Fig. S4), phospho-MCAK, HEC1 and the Aurora B complex (followed by survivin-GFP, Fig. 7A) after SMC2 depletion revealed that the phospho-S95 was markedly reduced between sister kinetochores, while survivin was often stretched along a much longer distance than in normal cells. Assaying S95 MCAK phosphorylation by quantitative immunoblotting after monastrol release showed that SMC2-depleted cells released for 30 min had a>2 fold decrease, relative to histone H3 phosphorylation on S10 (the latter is a marker for Aurora B activity on chromosome arms [46], [47]) (Fig. 7B). Analysis of a time-course following release from nocodazole into drug-free media or taxol, revealed that tension at kinetochores correlated with reduced Aurora B activity with respect to both MCAK (Fig. 7C) and CENP-A (Fig. 7D) phosphorylation. In the case of CENP-A, both constitutive (Fig. 7D, immunoblotting at time 0, also data not shown) and tension-specific (Fig. 7C, 30 min and micrograph arrow) decrease of Aurora B activity was reproducibly detected.

## Discussion

**Figure 8:**
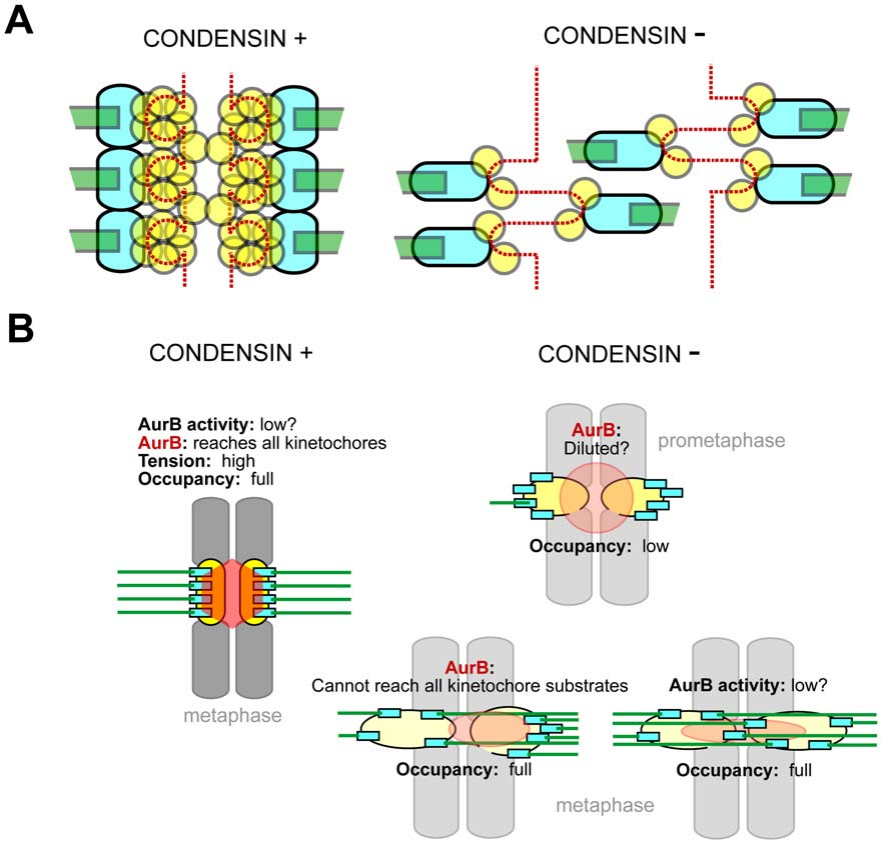
Models of the condensin-loss-mediated centromere and kinetochore dysfunction. A) Putative decondensation of centromeric chromatin resulting from the loss of condensin and CENPA. Once the centromeric chromatin (gray circles) becomes depleted and disorganized the individual microtubule-attachment sites in the kinetochore (blue ovals) become semi-independent and acquire more flexibility to attach to a microtubule (green) for the incorrect pole. B) Putative mechanisms of Aurora B inactivation at centromeres in condensin-depleted chromosomes. Left panel: Aurora B is properly localized and can reach its kinetochore substrates. Aurora B activity at centromeres is believed to decrease in normal cells once highest tension between sister kinetochores is achieved and sister kinetochores are stably localized at the spindle mid-zone [58], [59]. Color-coding as in (A), Aurora B – red polygon. Right top panel: Partial disconnect between Aurora B pool and kinetochores in prophase (and/or under nocodazole arrest) resulting in putative constitutive (tension-independent) decrease of Aurora B activity. Right bottom: putative disconnect between Aurora B and its target proteins (e.g. MCAK and HEC1) upon stretching of either asymmetrically or quasi-symmetrically (double-merotelic) mis-attached pair of centromeres/kinetochores.

We have shown that condensins I and II are required to prevent defects in centromeres derived from a combined loss of mitotic centromere rigidity and depletion of CENP-A (Fig. 8A). The resultant dispersed microtubule attachment sites produce excessive merotelic attachments and partial depletion at centromeres of the Aurora B activity that is needed to correct these improper attachments (Fig. 8B). Ultimately, despite an at least partially functioning kinetochore-dependent mitotic checkpoint, chromosomes enter anaphase with improperly attached kinetochores and chromosome bridges that break during (or before) cytokinesis.

The formation of such bridges has been commonly attributed to the inability of sister chromatid arms to disjoin in anaphase [1]. To the contrary, we have found that unresolved merotelic attachment, as opposed to continuing pairing of sister chromatids after anaphase onset, is the primary contributor to missegregation that is accompanied by DNA damage late in mitosis. The simultaneous microtubule-dependent stretching (Fig. 4) of centromeric proteins (CREST and CENP-A) and the proteins mediating microtubule-kinetochore contacts (BUB1 and HEC1) is as would be predicted for a substantial increase in merotely, accompanied by a disorganized midzone and asynchronous movement of kinetochores during anaphase (Fig. 5A). So too is a high incidence of bent and deformed kinetochore plates, which coincide with microtubules projecting to both poles (Fig. 5E, Fig. S3B). Combining all of this evidence, such attachment errors are almost certainly the actual cause of chromosomal bridges in anaphase in SMC2-depleted cells, because the “wait anaphase” signal of the mitotic checkpoint produced by unattached kinetochores is largely silenced by merotelic attachments [48], [49].

Our findings agree well with the hypothesis that relative impairment of bipolar attachment and successful segregation upon condensin dysfunction (i.e. decondensation of centromeric chromatin) is a function of the number of microtubule attachment sites (Fig. 8A) and size of kinetochores in different organisms. Indeed, in case of *C. elegans*, with its highly spread-out microtubule-attachment sites, merotelic attachments are easily detectable upon condensin inactivation [50]. Human kinetochores with 12–22 microtubule attachment sites [51] apparently fall into a similar category (this work as well as [6], [23]). At the same time, chicken cells, with notably fewer microtubules per kinetochore, show apparently “normal” kinetochores but substantially disrupted centromere structure [20], [31]. Finally, budding yeast kinetochores, each of which can capture only a single microtubule and thus have no possibility of merotely, show centromere chromatin stretching and disrupted composition, but kinetochores achieve proper bipolar orientation [18].

The results described here indicate that dysfunction of human condensin both compromises an important controlling mechanism (correction of syntelic and merotelic attachments) and leads to chromosome damage (breaks) and nondisjunction. Moreover, it appears that chromosomal damage directly results from centromere defects, not an independent non-disjoining defect at the chromosomal arms. Thus, a single hypomorphic condensin mutation can potentially generate a wide spectrum of genome instability signatures seen in cancers (a link of condensin to cancer was recently proposed in one case [52]).

### Condensin dependent CENP-A loading and kinetochore integrity

Using pulse-labeling of CENP-A, we have shown that human centromeres, like yeast counterparts, fail to load a correct level of new CENP-A in the absence of condensin (Fig. 3), which may be either a cause or a consequence of centromere stretching in metaphase (Fig. 1F). This depletion was unnoticed in prior work, probably because the long half life of CENP-A protein [29] was a confounding influence for conventional immunocytochemistry. Assuming that human kinetochores have a repeated-module (repeated loop) structure [53], [54], the loss of CENP-A would lead to increased centromere stretching by spindle forces, resulting in the loss of rigidity of kinetochores themselves, which was indeed observed (Fig. 4), and in the dispersion of microtubule attachment sites within the single kinetochore (Fig. 8A). Such disorganization would be expected to promote massive merotelic attachments, assuming that kinetochore propensity for microtubule binding itself is unaffected.

### Aurora B activity at kinetochores is compromised by condensin depletion

Aurora B activity in correcting syntelic and merotelic attachments [49], [55] goes hand in hand with the mitotic checkpoint in insuring faithful chromosome segregation. Compromise in Aurora B activity or condensin-deficiency results in the same (epistatic) missegregation. There are probably several mechanisms of Aurora B inactivation at condensin-depleted centromeres. First, there is a possibility that a constitutive decrease of Aurora B activity is characteristic for condensin-depleted cells (Fig. 6D: inability to recover from syntelic attachments). The constitutive decrease of p-CENP-A in nocodazole (Fig. 7D) argues in favor of such an interpretation. This effect may be due to the physical relaxation of a decondensed centromere, leading to physical separation of inner-centromere-anchored Aurora B from its substrates at centromeric chromatin (Fig. 8B, top panel). The second possibility is the failure to activate Aurora B at condensin-less centromeres due to stretching of its chromosome passenger scaffold and resulting “dilution” of Aurora B activity (Fig. 8B, lower panel; Fig. 6A right panel; Fig. 7D arrow) and/or further physical separation of Aurora B from its substrates at kinetochore plates [56]. The extreme case of such separation is exemplified by off-midzone centromeres indicated by arrows in Fig. 6A. Finally, Aurora B silencing at condensins-less merotelic kinetochores may be produced physiologically [57], [58], [59], due to dramatic increase in symmetrically double-merotelic sister kinetochore pairs, which would generate quasi-wild type tension and anaphase entry following mitotic checkpoint silencing. These scenarios are not mutually exclusive and can co-exist in the same cell. Indeed, our experiments following recovery of condensin depleted cells from monastrol and nocodazole treatments strongly argue that the specific inactivation of Aurora B at centromeres results from condensin loss. Whether this inactivation results from a direct biochemical signal from condensin loss, in addition to partial Aurora B delocalization and “disconnect” from its targets [56], remains to be elucidated.

## Materials and Methods

### SNAP-tagged CENP-A

Double thymidine synchronization, SNAP quenching and TMR labeling on HeLa cells stably expressing near endogenous levels of CENP-A-SNAP were performed as described [29]. 60 pM of GAPDH control siRNAs or SMC2 specific siRNAs were transfected using Oligofectamine (Invitrogen) during S phase, 4 hours after release from the first thymidine block. Following assembly of nascent TMR-labeled CENP-A-SNAP in early G1, cells were collected in thymidine at the end of G1, fixed and counter stained for CENP-C (gift from W. Earnshaw) and DAPI. Digital images were captured using a DeltaVision RT system (Applied Precision) controlling an interline charge-coupled device camera (Coolsnap; Roper) mounted on an inverted microscope (IX-70; Olympus). For quantification over 300 cells were imaged per condition at 1×binning using a 40×oil objective at 0.6 µm z sections spanning the entire nucleus. Maximum signals were projected as 2D images using softWoRx (Applied Precision) and converted to unscaled TIFF images. Maximum intensity per nucleus was determined and background subtracted using MetaMorph (Molecular Devices). Images shown in Fig. 3C were captured using a 100X objective at 0.2 µm z sections, deconvolved and projected using softWoRx.

### Antibodies

The following antibodies were used: polyclonal anti-xBUB1 [32]; human monoclonal anti-CENP-A (a kind gift from K. Yoda, Nagoya University, Nagoya, Japan) [60]; phosphorylated histone H3 S10 (Millipore), rabbit anti-hMAD2 (Berkeley antibody company, Richmond, CA); anti-cyclin B1 and anti-CDC2 (Santa Cruz Biotechnology); anti–α-tubulin and anti-γ-tubulin antibodies (Sigma-Aldrich); anti–pH2AX (GeneTex); anti-hAurora B (BD Transduction Laboratories) and human CREST sera (gift from W. Brinkley). The antibodies against xSMC2 (C-SKTKERRNRMEVDK), hCAP-D2 (C-DETPKKTTPILRASARRHRS), hCAP-D3 (C-SRRSLRKTPLKTAN), HEC1 (aa 77-193 of xHec1), hMCAK, phospho-hMCAK(S95; C-IQKQKRR(pS)VNSKIPA), hSMC2 (C-CSTVARFTQCQNGKISKEAKSKAKPPKGAHVEV) were generated in rabbits and affinity purified. All secondary antibodies were conjugated to Alexa Fluor −488, −594, −647 or 750 were from Molecular Probes (Eugene).

### Quantitative immunoblotting analysis

Both blotting and imaging with the Odyssey imaging system (LI-COR, Lincoln, NE) were performed by following the manufacturer's protocols. The secondary antibodies included IRDye 700 and 800 (1∶5000; Rockland Immunochemicals, Gilbertsville, PA). Membranes were scanned at 700 nm and 800 nm simultaneously with an Odyssey instrument at 169 µm resolution, medium or high quality, focus offset 0.0 mm and intensity setting of 6 for the 700 nm channel and 4 for 800 nm channel. Normalization of gel bands was accomplished by analysis of two wavelengths, where α-tubulin, CDC-2 or phospho-histone H3 was used as loading control. All signaling data are plotted±SEM, with N = 3 or 4.

### Cell culture and siRNA

HeLa, HeLa^H2B−GFP^ [61] and HeLa^Survivin-EGFP^ [32] cells were grown in DMEM supplemented with 10% FBS (Gibco, Invitrogen). Cells were transfected with the siRNA duplexes (Qiagen) designed to repress hSMC2 (#1 5′-AATGCTATCACTGGCTTAAAT-3′ or #2 5′-AATGTTGTAGTAGACACAGAA-3′); hCAP-D2 and hCAP-D3 subunits as in [4] and mismatched oligonucleotide (5′-AATGTGCGACCTACTCAGATT-3′) as a negative control using Oligofectamine-2000 and serum-free OptiMEM (Invitrogen), according to the manufacture's instructions.

### Complementation of condensin depletion

The GAACGACTCTACAATGTTGTtGTAGAtACAGAAGTTACTGGTAAAAAG oligonucleotide was used to mutagenize full-length *SMC2* cDNA (a gift from K. Yokomori), which was subsequently inserted into NheI and XhoI sites of pcDNA3.1, to produce the pcDNA3.1/SMC2-2 complementation plasmid, resistant to RNAi with RNA duplex #2, due to silent mismatch mutation at the siRNA-corresponding site. HeLa cells were transfected by pcDNA3.1/SMC2-2 (4–5 µg DNA per 6 cm dish) mixed with pEGFP-N3 plasmid (Clontech, USA) as a transfection marker 48 h after hSMC2 siRNA #2 transfection. The DNA transfection mix contained pcDNA3.1/SMC2-2 (4–5 µg DNA per 6 cm dish), pEGFP-N3 (1 µg), 10 µl PromoFectin (PromoKine, Germany) and serum-free medium. After a period of 24 h or 48 h cells were collected for the Western-blotting and fixed for immunofluorescence microscopy.

### Release of cells from nocodazole block

For the time-course nocodazole-release experiments, following 48 h mock- or hSMC2 siRNA transfection, HeLa cells were arrested for 16 h with 0.3 µg/ml nocodazole. Mitotic cells were collected, washed 3 times in fresh culture medium and released into either drug-free medium (control) or medium containing 1 µg/ml Paclitaxel (Calbiochem), or medium containing nocodazole (0.3 µg/ml). Cells were collected at indicated time points, washed in PBS and lysed on ice for 5 min in buffer A (0.8x CSF-XB buffer [32] containing 0.2% Triton X-100, 100 nM okadaic acid and 0.2 mM AEBSF) by passing through 25 g needle. Samples were prepared by mixing cell lysates with SDS-PAGE sample buffer. For chromatin isolation, cell lysates were layered over 1 ml of cushion (buffer A containing 30% glycerol) and spun down at 10 000 g for 5 min in swinging bucket Eppendorf rotor. Pellets were resuspended in SDS-PAGE sample buffer, boiled for 10 min and extensively vortexed to fragment chromatin.

### Immunofluorescence microscopy

Cells on coverslips were processed for immunofluorescence microscopy 48 or 72 h after siRNA transfection. Cells were either left untreated or incubated with 0.4 µg/ml nocodazole for 16 h to induce mitotic arrest. Alternatively, cells were treated with 20 µM monastrol (Tocris) for 4 h and released into media containing 10 µM MG132 (Calbiochem) or 10 µM MG132 and 10 nM ZM 447439 (Tocris) together for 2 h before fixing. Cells were washed with PBS and immediately fixed with 4% paraformaldehyde, or washed in BRB 80 buffer (100 mM Pipes, pH 6.8, 5 mM EGTA, 2 mM MgCl_2_, 1 mM DTT, 250 mM sucrose), containing 0.5% Triton-X100 for 3 min at 23°C followed by fixation with 4% paraformaldehyde. After fixation, cells were washed three times in TBS-T, permeabilized with 0.25% Triton-X100 and blocked with 5% BSA for 30 min followed by staining with corresponded primary antibody for 40 min at RT or overnight at 4°C, followed by staining with corresponding secondary antibodies for 40 min. Samples were counterstained with Hoechst 33342 (Sigma-Aldrich) and mounted in Vectashield anti-fade mounting medium (Vector Laboratories). Slides were examined with ZEISS Axioskop or Axiovert fluorescence microscopes and images were collected and analyzed with OpenLab software.

### Deconvolution microscopy

Images of stained cells were acquired on a deconvolution microscope (DeltaVision; Applied Precision) equipped with a CCD camera (CoolSNAP; Roper Scientific). This system consists of an inverted microscope IX70 (Olympus) with a 1.35 NA 100x objective and FITC, Rhodamine, and Cy5 filter sets, a Photometrics CH350 12-bit camera (Photometrics) with a KAF1400 chip, and a UNIX-based Silicon Graphics O2 workstation with SoftWoRx software installed. Camera wells were not binned, yielding a pixel size of 0.07 µm in x and y. z steps were set to 0.07 µm, yielding cubic voxels. Image sizes in x, y, and z were 512×512×140–200. 30–80 z sections were acquired at 0.2-µm steps and images were deconvolved with the SoftWoRx software package (Applied Precision) using Decon3d with the default settings and experimental point spread function (PSF).

### Live imaging

Asynchronous HeLa cells stably expressing H2B−GFP were grown on LabTekTM chambers (Nunc, Naperville, IL) in culture medium containing 10% fetal bovine serum and transfected with the siRNA as described. Imaging was performed after 24–48 hours of oligonucleotide transfection on a spinning disk confocal microscope (Ultraview ERS, Perkin-Elmer, Boston, MA) equipped with an environmental chamber and a 1.4 NA 63x objective. 14% power in the 488-nm line was applied. Stacks of 20 images were taken at 2-min intervals.

### Transmission electron microscopy

For EM experiments cells were grown on poly-L-lysine–coated ACLAR coverslips (Ted Pella, Inc.) and fixed at room temperature with 2.5% glutaraldehyde in 0.1 M phosphate buffer, pH 7.4 for 30 min. Alternatively, mitotic cells were collected by shake-off and fixed as above. The coverslips or cells pellet were washed twice in phosphate buffer and then post-fixed in 1% aqueous OsO_4_ for 60 min at 4°C followed by quick rinse in double distilled water, ethanol dehydration series up to 100% ethanol, and by a Embed-812 resin (Electron Microscopy Sciences) infiltration series up to 100% resin. The epoxy resin was polymerized for 20 hours in an oven set at 60°C. Around 90-nm thin sections were prepared on a Reichert-Jung Ultracut-E ultramicrotome, collected and transferred to 300-mesh EM grids using Perfect Loop (DiATOME). The sections were post-stained with uranyl acetate and lead citrate and examined in a JEOL 1010 transmission electron microscope operating at 80 kV.

## Supporting Information

Figure S1 Depletion of either condensin I or II results in abnormal centromere structure. A) CAP-D2 and CAP-D3 depletion by RNAi. Immunoblotting to determine CAP-D2 or CAP-D3 levels detected with specific antibodies (see Methods) three days after siRNA transfection; α-tubulin - loading control; mock - mismatched CAP-D3 RNAi. Loss of either protein was confirmed by indirect immunofluorescent staining of fixed HeLa cells (not shown). B) Metaphase centromere defects in condensins-depleted cells HeLa cells were fixed three days after transfection with CAP-D2 or CAP-D3 siRNA or corresponding mismatched siRNA (CAP-D3 mis RNAi is shown). Metaphases were identified by DAPI (DNA) staining and spindle morphology (anti-alpha-tubulin antibodies). CREST antibody staining of centromeres shows both diffuse metaphase plates and failure of sister centromeres to separate (inserts).
(4.34 MB TIF)

Figure S2 Condensin-depleted chromosomes break in late anaphase. Asynchronous HeLa cells were transfected with either specific SMC2 siRNA (SMC2 RNAi) or the control mismatched siRNA (mis RNAi). The fixed cells were stained with phosphorylated H2AX (γ-H2AX) and CREST antibodies and DNA was stained with Hoechst 33342. Metaphase (A) and early anaphase (B) condensin-depleted chromosomes are free from double strand breaks, as indicated by diffuse staining with γ-H2AX antibodies. Only telophase, but not early anaphase, cells display a high incidence of double strand breaks.
(3.56 MB TIF)

Figure S3 Condensin depletion results in extremely stretched centromeric chromatin disrupting kinetochore morphology identifiable by EM. A) The CREST immuno-gold EM staining of control and SMC2-depleted centromeres is shown. The extreme stretching of most centromeres is likely accompanied by a deformed/disrupted kinetochore structure, which precludes identification of most merotelic kihetochores by EM in fig. 5C–E. Pre-embedding immunocytochemistry procedures were made as following: cells were fixed with a mixture of 2% paraformaldehyde and 0.5% glutaraldehyde (EMS, Fort Washington, PA) in 0.1 M phosphate buffer at pH 7.4 for 45–60 min, washed with buffer, permeabilized with 0.1% saponin and blocked with 5% normal goat serum in PBS for 1 hr, incubated with the CREST antibody for 1 h, washed, incubated with the secondary goat-anti-human antibody conjugated to 1.4 nm gold (1∶250; Nanogold from Nanoprobes, Yaphand, NY) for 1 hr, washed and fixed with 2% glutaraldehyde in PBS for 1 h. Samples were then washed in water, silver enhanced (HQ 2012 silver enhancement kit, Nanoprobes) following manufacturer instructions, treated with 0.2% OsO4 in buffer for 30 min and with 0.25% uranyl acetate in buffer overnight at 4 C, washed and dehydrated in ethanol and finally embedded in epoxy resins as described in Methods. Controls were done by omitting primary antibodies. B) Merotelic mis-attachment potentially leads to disruption of kinetochore structure in condensin-depleted human cells. Examples of more severe (than in Fig. 5) merotelic mis-attachments of kinetochores in hSMC2-depleted cells are shown.
(9.13 MB TIF)

Figure S4 Condensin depletion does not impair MCAK loading. Control and SMC2-depleted cells were double-stained with Aurora B and MCAK antibodies 20 min after release from nocodazole arrest (procedure - as in Fig. 6C).
(1.51 MB TIF)
