# Supplementary figures and images for: Human Condensin Function Is Essential for Centromeric Chromatin Assembly and Proper Sister Kinetochore Orientation
Source: PLoS One. 2009 Aug 28;4(8):e6831. doi: 10.1371/journal.pone.0006831 (PMC2730017; doi:10.1371/journal.pone.0006831)

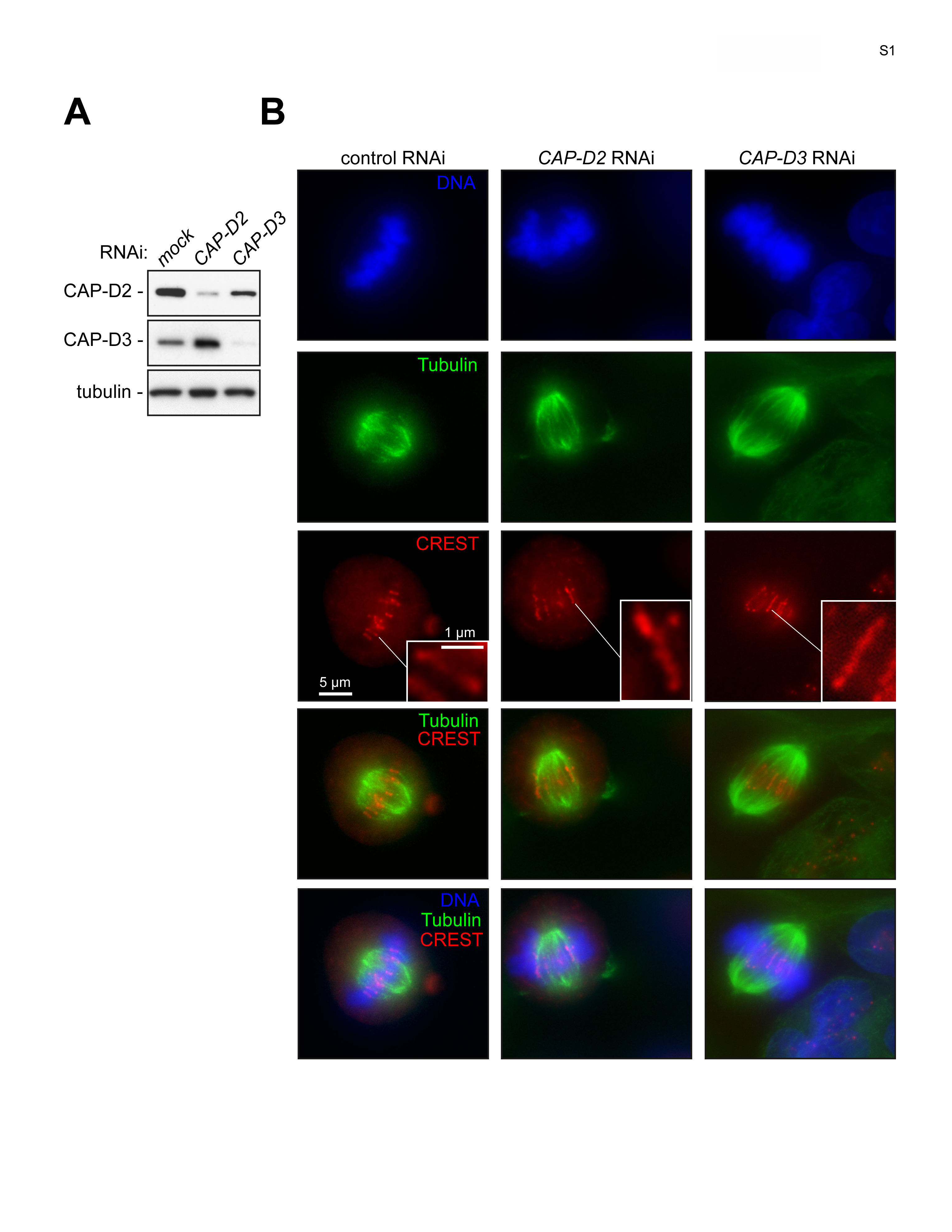

Supplement: Figure S1 — Depletion of either condensin I or II results in abnormal centromere structure. A) CAP-D2 and CAP-D3 depletion by RNAi. Immunoblotting to determine CAP-D2 or CAP-D3 levels detected with specific antibodies (see Methods) three days after siRNA transfection; α-tubulin - loading control; mock - mismatched CAP-D3 RNAi. Loss of either protein was confirmed by indirect immunofluorescent staining of fixed HeLa cells (not shown). B) Metaphase centromere defects in condensins-depleted cells HeLa cells were fixed three days after transfection with CAP-D2 or CAP-D3 siRNA or corresponding mismatched siRNA (CAP-D3 mis RNAi is shown). Metaphases were identified by DAPI (DNA) staining and spindle morphology (anti-alpha-tubulin antibodies). CREST antibody staining of centromeres shows both diffuse metaphase plates and failure of sister centromeres to separate (inserts). (4.34 MB TIF) [file pone.0006831.s001.tif]

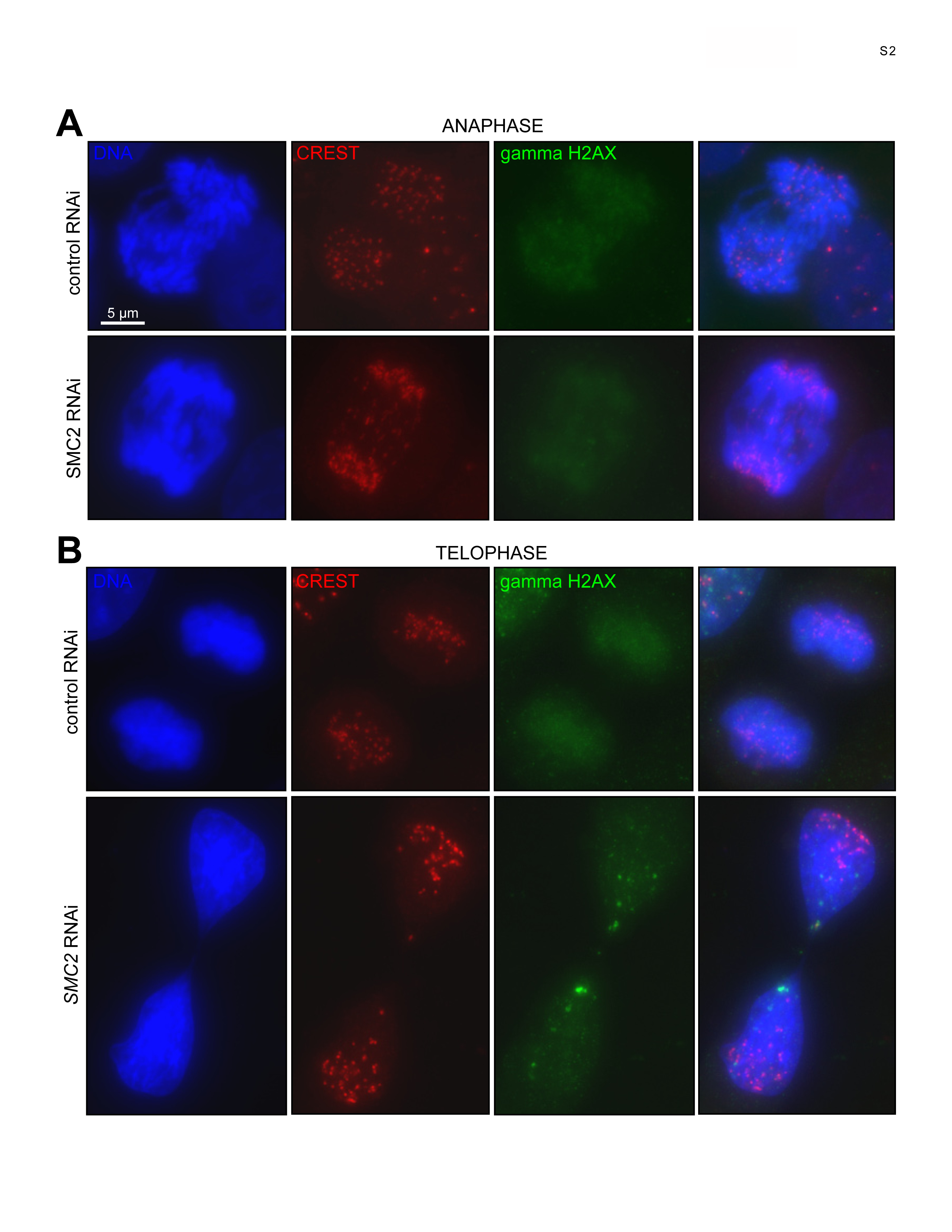

Supplement: Figure S2 — Condensin-depleted chromosomes break in late anaphase. Asynchronous HeLa cells were transfected with either specific SMC2 siRNA (SMC2 RNAi) or the control mismatched siRNA (mis RNAi). The fixed cells were stained with phosphorylated H2AX (γ-H2AX) and CREST antibodies and DNA was stained with Hoechst 33342. Metaphase (A) and early anaphase (B) condensin-depleted chromosomes are free from double strand breaks, as indicated by diffuse staining with γ-H2AX antibodies. Only telophase, but not early anaphase, cells display a high incidence of double strand breaks. (3.56 MB TIF) [file pone.0006831.s002.tif]

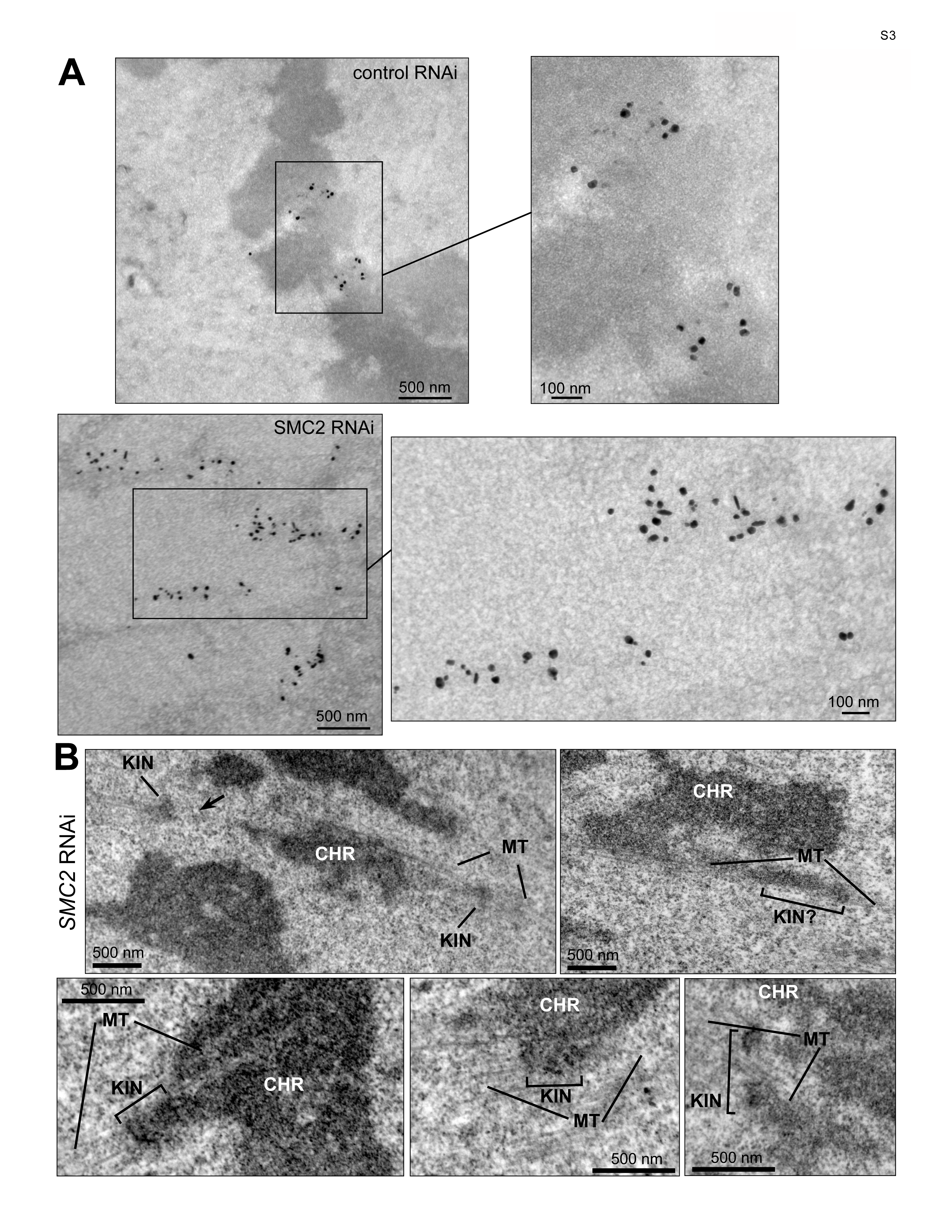

Supplement: Figure S3 — Condensin depletion results in extremely stretched centromeric chromatin disrupting kinetochore morphology identifiable by EM. A) The CREST immuno-gold EM staining of control and SMC2-depleted centromeres is shown. The extreme stretching of most centromeres is likely accompanied by a deformed/disrupted kinetochore structure, which precludes identification of most merotelic kihetochores by EM in fig. 5C–E. Pre-embedding immunocytochemistry procedures were made as following: cells were fixed with a mixture of 2% paraformaldehyde and 0.5% glutaraldehyde (EMS, Fort Washington, PA) in 0.1 M phosphate buffer at pH 7.4 for 45–60 min, washed with buffer, permeabilized with 0.1% saponin and blocked with 5% normal goat serum in PBS for 1 hr, incubated with the CREST antibody for 1 h, washed, incubated with the secondary goat-anti-human antibody conjugated to 1.4 nm gold (1∶250; Nanogold from Nanoprobes, Yaphand, NY) for 1 hr, washed and fixed with 2% glutaraldehyde in PBS for 1 h. Samples were then washed in water, silver enhanced (HQ 2012 silver enhancement kit, Nanoprobes) following manufacturer instructions, treated with 0.2% OsO4 in buffer for 30 min and with 0.25% uranyl acetate in buffer overnight at 4 C, washed and dehydrated in ethanol and finally embedded in epoxy resins as described in Methods. Controls were done by omitting primary antibodies. B) Merotelic mis-attachment potentially leads to disruption of kinetochore structure in condensin-depleted human cells. Examples of more severe (than in Fig. 5) merotelic mis-attachments of kinetochores in hSMC2-depleted cells are shown. (9.13 MB TIF) [file pone.0006831.s003.tif]

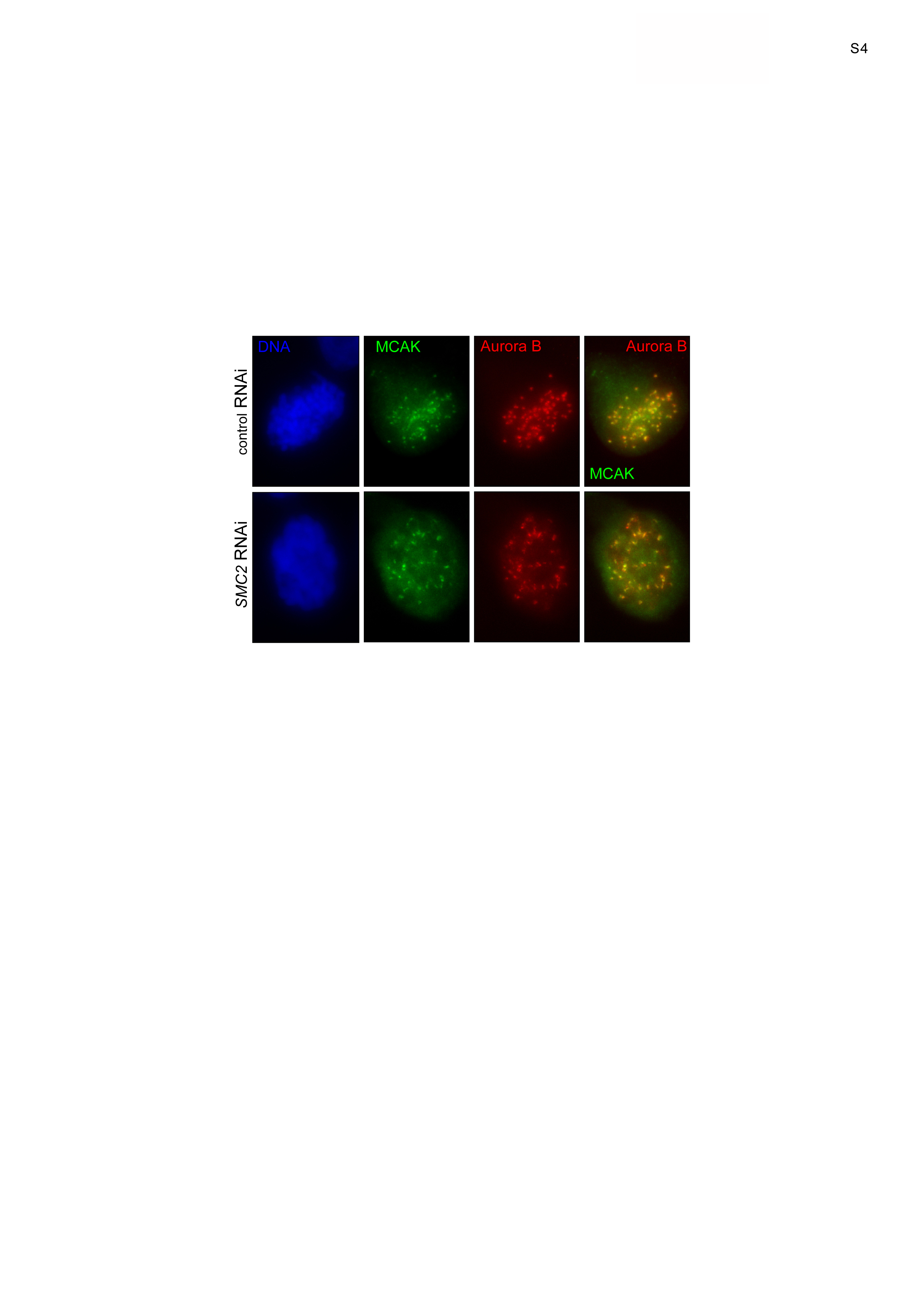

Supplement: Figure S4 — Condensin depletion does not impair MCAK loading. Control and SMC2-depleted cells were double-stained with Aurora B and MCAK antibodies 20 min after release from nocodazole arrest (procedure - as in Fig. 6C). (1.51 MB TIF) [file pone.0006831.s004.tif]
